# Association of oily fish and nonoily fish intakes with all-cause mortality and cause-specific mortality: a large population-based prospective study

**DOI:** 10.1186/s12967-023-04097-4

**Published:** 2023-04-26

**Authors:** Jian Zhou, Xiankui Tan, Yihan Li, Rongjun Wan, Ziyi Wu, Zhengjun Lin, Wanchun Wang, Yingquan Luo, Tang Liu

**Affiliations:** 1 Department of Orthopedics, The Second Xiangya Hospital of Central South University, Renmin Middle Road No. 139, Changsha, 410011 Hunan China; 2 Research Center of Ultrasonography, The Second Xiangya Hospital of Central South University, Changsha, 410011 Hunan China; 3 Department of Respiratory Medicine, National Key Clinical Specialty, Branch of National Clinical Research Center for Respiratory Disease, Xiangya Hospital, Central South University, Changsha, 410008 Hunan China; 4 Laboratory of Bone Disorder, The Second Xiangya Hospital of Central South University, Changsha, 410011 Hunan China; 5 Department of General Medicine, The Second Xiangya Hospital of Central South University, Renmin Middle Road No. 139, Changsha, 410011 Hunan China

**Keywords:** Oily fish, Nonoily fish, All-cause mortality, Cause-specific mortality, UK Biobank

## Abstract

**Background:**

There are inconsistent results of cohort studies analyzing the association between fish intake and mortality.

**Objective:**

This study was performed to explore the association of oily fish consumption and nonoily fish consumption with all-cause mortality and cause-specific mortality.

**Methods:**

A total of 431,062 participants from the UK Biobank who were without cancer or cardiovascular disease (CVD) at baseline between 2006 and 2010 were included in this study, and they were followed up through 2021. We constructed Cox proportional hazard models to calculate the hazard ratio (HR) and 95% confidence interval (CI) to assess the correlation of oily fish and nonoily fish intakes with mortality. Then, we performed subgroup analyses, and sensitivity analyses were developed and performed to examine the robustness of this study.

**Results:**

Among the participants, 383,248 (88.9%) and 410,499 (95.2%) consumed oily fish and nonoily fish, respectively. Compared with the participants who did not consume oily fish, the adjusted HRs for the association of oily fish consumption (1 serving/week) with all-cause mortality and CVD mortality were 0.93 (0.87 to 0.98; *p <* 0.05) and 0.85 (0.74 to 0.98; *p <* 0.05), respectively. The multivariable-adjusted HRs of all-cause mortality for those who reported consuming < 1 serving/week of oily fish were 0.92 (0.86 to 0.98; *p <* 0.05).

**Conclusion:**

Compared with participants who reported never consuming oily fish, the consumption of oily fish with 1 serving/week was more beneficial for all-cause and CVD mortality.

**Supplementary Information:**

The online version contains supplementary material available at 10.1186/s12967-023-04097-4.

## Introduction

Healthy lifestyles including a well-balanced diet are beneficial for preventing noncommunicable diseases, such as cancer and cardiovascular disease (CVD) [1, 2]. Fish is generally considered to be a nutrient-dense food because it contains several nutrients [3, 4]. Dietary guidelines in several countries recommend increasing the consumption of fish, especially oily fish, as an important part of a healthy eating pattern [5]. Fish provides the long-chain omega-3 fatty acids eicosapentaenoic acid and docosahexaenoic acid, which have been shown to have benefits on cardiovascular health [6–8]. Currently, the association of omega-3 supplementation with CVD outcomes remains controversial. Two meta-analyses demonstrated that omega-3 supplementation was not associated with all-cause mortality and cardiovascular outcomes [9, 10]. In contrast, another published meta-analysis [11] including three new trials [12–14] indicated that omega-3 supplementation provided a significant benefit to the risk of CVD outcomes. Additionally, evidence from previous epidemiological studies had inconsistent results on the association between fish intake and all-cause mortality [15–20]. Moreover, it remains uncertain whether a consumption of fish higher than the recommended level is associated with higher risks of all-cause mortality and cause-specific mortality. Therefore, more large-scale cohort studies on fish consumption and clinical outcomes, especially studies that differentiate between oily fish and nonoily fish, are still needed.

To further understand the association between fish consumption and mortality, we performed a large population-based cohort study. A total of 431,062 participants from the UK Biobank were enrolled in this study to explore the relationship of oily fish and nonoily fish intakes with all-cause and cause-specific mortality, and the relevant correction factors were also investigated.

## Methods

### Participants and study design

**Fig. 1:**
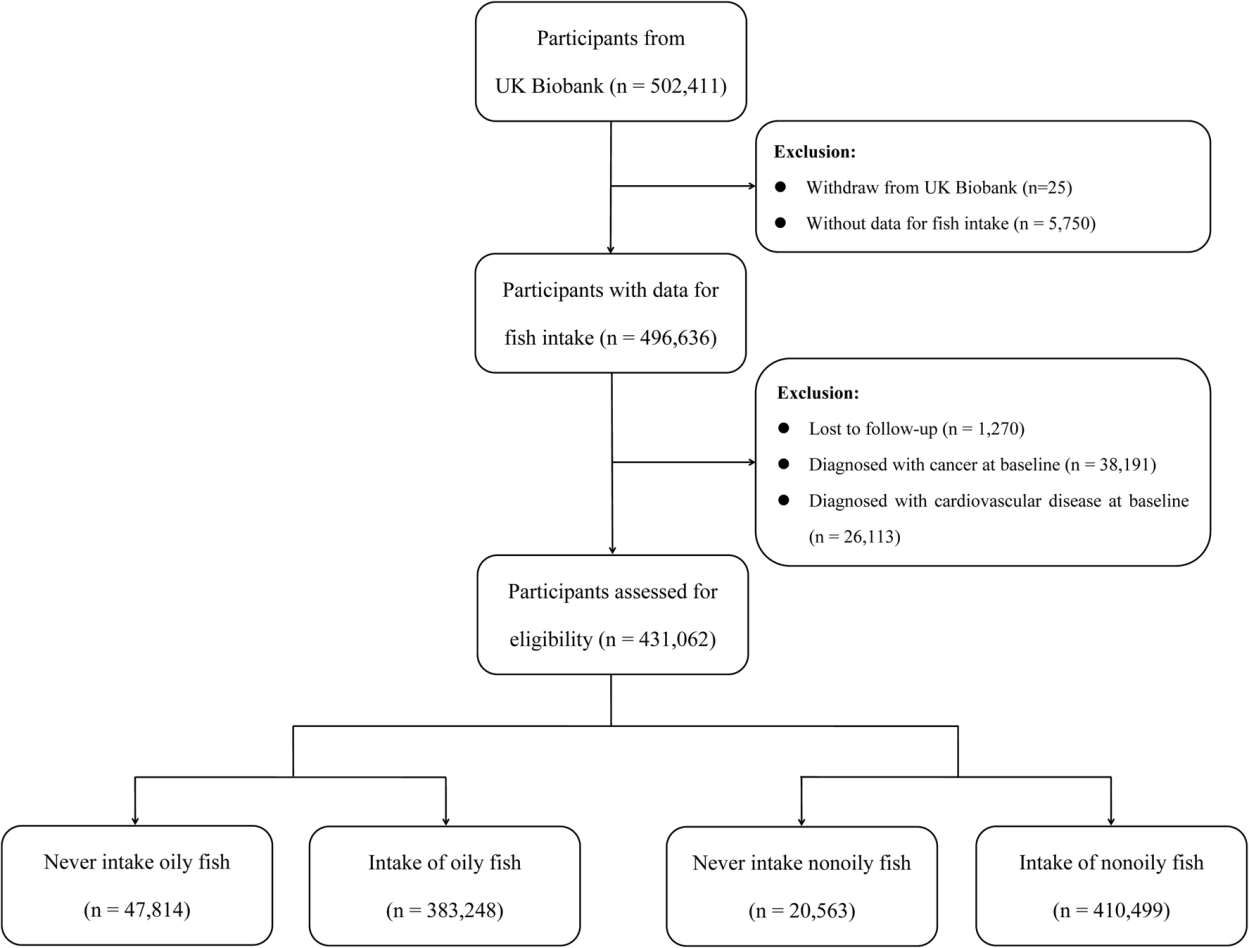
Flowchart of participants enrolment

The UK Biobank collected disease and lifestyle information and genotype data from approximately 500,000 participants aged 40 to 69 years from England, Scotland, or Wales between March 2006 and July 2010. The full UK Biobank study protocol is available at https://www.ukbiobank.ac.uk/media/gnkeyh2q/study-rationale.pdf. In the present study, a total of 502,411 participants from the UK Biobank were recruited. We excluded 25 participants who withdrew from the UK Biobank project and 5750 participants without data on fish consumption. After excluding these participants, 1270 participants who were lost to follow-up were also excluded. Then, 38,191 participants diagnosed with cancer at baseline and 26,113 participants diagnosed with CVD at baseline were excluded. Finally, a total of 431,062 participants without cancer or CVD at baseline were included in the present study (Fig. 1).

### Exposure assessment

All participants from the UK Biobank project were asked to finish a questionnaire using a touchscreen device. To collect information on oily fish intake, the question, “How often do you eat oily fish? (e.g., sardines, salmon, mackerel, and herring),” was asked. To collect information on nonoily fish intake, the question, “How often do you eat other types of fish? (e.g., cod, tinned tuna, and haddock),” was asked. Then, the participants selected answers from a numbered scale, including 0: never, 1: less than once a week, 2: once a week, 3: 2–4 times a week, 4: 5–6 times a week, 5: once or more daily, − 1: do not know, and − 3: prefer not to answer (Additional file 1: Table S1).

### Assessment of outcomes

The main outcomes of this study were all-cause mortality, cancer mortality, and CVD. We obtained the cause and date of death via linking to the Death Registry of the National Health Service (NHS) Information Centre for the UK and Welsh participants and via linking to the Death Registry of the Scottish NHS Central Registry for the Scottish participants. The detailed information of these death registries is accessible at http://content.digital.nhs.uk/services. We adopted this date as the end date for follow-up, unless death occurred. The outcomes were classified by the *International Classification of Diseases, 10th Revision* (ICD 10). In the present study, we analyzed all-cause mortality, cancer mortality (codes C00 to D48), and CVD mortality (codes I00 to I99) [21–24].

### Other variables

According to the protocol of the UK Biobank, questionnaires were administered to obtain information on several variables related to sociodemographic factors, socioeconomic status, lifestyle habits, health condition, and supplementation. The sociodemographic factors assessed included age, sex and ethnic background. The socioeconomic status factors assessed included the Townsend Deprivation Index score. The lifestyle habits assessed included physical activity, smoking status, alcohol consumption, processed meat intake, red meat intake, fruit intake, vegetable intake, tea intake and variation in diet. The health conditions assessed included body mass index (BMI), hypertension and diabetes. The variables assessed for supplementation included the use of fish oil. We calculated BMI as the weight (kg) divided by the height (m) squared. Hypertension was defined as a diagnosis as hypertension by doctor, a systolic blood pressure of ≥ 140 mmHg or a diastolic blood pressure of ≥ 90 mmHg or taking blood pressure medication. Diabetes were diagnosed by a physician. Participants were divided into two groups according to the total moderate physical activity minutes each week (< 150 or ≥ 150 min/week) and the 1 min with vigorous physical activity equals 2 min with moderate physical activity. The other information was collected from the UK Biobank (www.ukbiobank.ac.uk).

### Statistical analysis

We performed Cox regression models, with two models constructed to explore the association of fish intake with all-cause mortality and cause-specific mortality. First, we constructed a basic model adjusting for baseline age and sex (male or female). After that, the multivariable model was further adjusted for ethnic background (white or others), Townsend Deprivation Index, body mass index, physical activity (< 150 or ≥ 150 min/week), smoking status (current smoking or other), alcohol intake, processed meat intake, poultry intake, beef intake, lamb/mutton intake, pork intake, hypertension (yes or no), diabetes (yes or no), fish oil use (yes or no), fruit consumption, vegetables consumption, tea intake, variation in diet (yes or no). Oily fish and nonoily fish were mutually adjusted for each other.

Additionally, we performed several subgroup analyses, including ones by age (≥ 60 years vs. <60 years), sex (male vs. female), ethnic background (white vs. others), Townsend Deprivation Index (greater than or equal to average vs. less than average), smoking status (current vs. other), alcohol intake (≥ 3 times/week vs. <3 times/week), BMI (≥ 30 kg/m^2^ vs. <30 kg/m^2^), hypertension (yes vs. no), diabetes (yes vs. no), physical activity (≥ 150 min/week vs. <150 min/week), fish oil supplementation (yes vs. no), processed meat intake (≥ 2 serv/week vs. <2 serv/week), and tea intake (≥ 4 drinks/day vs. <4 drinks/day).

### Sensitivity analysis

We performed sensitivity analyses to detect the stability of the results from the Cox proportional hazard models. First, we excluded the participants who died in the first 2 years of the follow-up. Second, we excluded the participants who used vitamin or mineral supplements. Third, we excluded the participants with a considerable variation in diet from week to week. All results from our study were assessed by calculating the hazard ratio (HR) and corresponding 95% confidence interval (CI). SAS version 9.4 (SAS Institute, Cary, NC), was used to conduct the analysis. A P value of < 0.05 was used to define statistically significant differences.

## Results

### Basic features of participants

The results revealed that those with a high intake of oily fish were older, more likely to be female, with more physical activity time per week, more likely to drink alcohol, more likely to have hypertension, and more likely to use fish oil and consume fruit and vegetable compared with the participants with a lower intake. The baseline features of participants included in this study are shown in Table 1.

**Table 1:** Baseline characteristics of participants

Characteristics	Oily fish intake, servings/week				Nonoily fish intake, servings/week			
	Never	< 1	1	≥ 2	Never	< 1	1	≥ 2
Participants, n (%)	47,814 (11.09)	145,157 (33.67)	162,517 (37.70)	75,574 (17.53)	20,563 (4.77)	126,431 (29.33)	213,976 (49.64)	70,092 (16.26)
Mean age (SD), y	53.61 (8.12)	54.98 (8.03)	56.7 (7.98)	57.73 (7.86)	53.63 (8.21)	55.21 (8.05)	56.54 (8.03)	56.2 (8.13)
Female, n (%)	25,438 (53.20)	76,522 (52.72)	92,398 (56.85)	42,938 (56.82)	11,666 (56.73)	67,826 (53.65)	118,025 (55.16)	39,779 (56.75)
Ethnic background, n (%)								
White	43,811 (91.97)	132,124 (91.32)	147,613 (91.10)	67,153 (89.26)	18,120 (88.60)	113,186 (89.85)	195,971 (91.86)	63,424 (90.82)
Other	3824 (8.03)	12,558 (8.68)	14,424 (8.90)	8079 (10.74)	2331 (11.40)	12,783 (10.15)	17,359 (8.14)	6412 (9.18)
Mean Townsend deprivation index (SD)	1.98 (1.71)	2 (1.48)	2.3 (1.52)	2.68 (1.76)	2.24 (1.86)	2.06 (1.55)	2.24 (1.54)	2.5 (1.72)
Mean BMI (SD), kg/m2	27.69 (5.15)	27.39 (4.79)	27.17 (4.61)	27.2 (4.69)	26.91 (5.06)	27.36 (4.80)	27.27 (4.66)	27.44 (4.83)
Physical activity (min/week)								
≥ 150	23,094 (67.22)	73,158 (65.46)	91,292 (69.19)	46,411 (73.91)	10,294 (67.66)	63,700 (65.94)	118,495 (68.90)	41,466 (72.69)
< 150	11,262 (32.78)	38,608 (34.54)	40,644 (30.81)	16,385 (26.09)	4921 (32.34)	32,902 (34.06)	53,497 (31.10)	15,579 (27.31)
Smoking status, n (%)								
Current	7079 (14.87)	16,138 (11.15)	14,646 (9.04)	6912 (9.18)	2641 (12.91)	14,279 (11.33)	20,791 (9.75)	7064 (10.12)
Other	40,529 (85.13)	128,624 (88.85)	147,316 (90.96)	68,382 (90.82)	17,818 (87.09)	111,768 (88.67)	192,515 (90.25)	62,750 (89.88)
Alcohol intake (times/week), n (%)								
≥ 3	14,883 (31.17)	62,538 (43.11)	76,561 (47.14)	35,210 (46.64)	6399 (31.17)	53,141 (42.06)	97,913 (45.79)	31,739 (45.33)
< 3	32,872 (68.83)	82,525 (56.89)	85,852 (52.86)	40,286 (53.36)	14,128 (68.83)	73,200 (57.94)	115,923 (54.21)	38,284 (54.67)
Processed meat intake (times/week), n (%)								
≥ 2	16,155 (33.86)	49,852 (34.39)	48,208 (29.70)	18,808 (24.92)	4555 (22.22)	38,407 (30.42)	68,433 (32.02)	21,628 (30.90)
< 2	31,550 (66.14)	95,123 (65.61)	114,113 (70.30)	56,655 (75.08)	15,946 (77.78)	87,849 (69.58)	145,280 (67.98)	48,366 (69.10)
Poultry intake(times/week), n (%)								
≥ 2	19,395 (40.68)	69,266 (47.77)	82,262 (50.67)	39,333 (52.09)	5737 (28.00)	57,135 (45.25)	105,385 (49.30)	39,831 (56.88)
< 2	28,284 (59.32)	75,747 (52.23)	80,094 (49.33)	36,176 (47.91)	14,753 (72.00)	69,143 (54.75)	108,377 (50.70)	30,196 (43.12)
Beef intake (times/week), n (%)								
≥ 2	5663 (11.91)	18,514 (12.79)	17,819 (11.00)	7082 (9.40)	1951 (9.53)	14,992 (11.90)	24,342 (11.41)	7793 (11.15)
< 2	41,894 (88.09)	126,243 (87.21)	144,218 (89.00)	68,252 (90.60)	18,515 (90.47)	111,007 (88.10)	189,003 (88.59)	62,082 (88.85)
Lamb/mutton intake (times/week), n (%)								
≥ 2	1190 (2.51)	4457 (3.08)	4521 (2.80)	2602 (3.46)	581 (2.84)	3962 (3.15)	5726 (2.69)	2501 (3.59)
< 2	46,269 (97.49)	140,059 (96.92)	157,220 (97.20)	72,559 (96.54)	19,849 (97.16)	121,819 (96.85)	207,184 (97.31)	67,255 (96.41)
Pork intake (times/week), n (%)								
≥ 2	1802 (3.8)	5594 (3.87)	5034 (3.11)	2639 (3.51)	658 (3.22)	4865 (3.87)	6889 (3.23)	2657 (3.81)
< 2	45,673 (96.2)	138,971 (96.13)	156,775 (96.89)	72,543 (96.49)	19,795 (96.78)	120,949 (96.13)	206,113 (96.77)	67,105 (96.19)
Hypertension, n (%)	25,865 (54.1)	79,066 (54.47)	93,661 (57.64)	45,551 (60.28)	10,461 (50.88)	68,773 (54.4)	123,861 (57.89)	41,048 (58.56)
Diabetes, n (%)	2506 (5.26)	6038 (4.17)	6824 (4.21)	3830 (5.08)	1130 (5.53)	5441 (4.32)	9349 (4.38)	3278 (4.69)
Fish oil use, n (%)	9842 (20.81)	38,862 (27.02)	55,224 (34.32)	29,607 (39.60)	3729 (18.39)	35,765 (28.57)	69,993 (33.01)	24,048 (34.68)
Mean fruit intake (SD), servings/d	1.98 (1.71)	2 (1.48)	2.3 (1.52)	2.68 (1.76)	2.24 (1.86)	2.06 (1.55)	2.24 (1.54)	2.5 (1.72)
Mean vegetable intake (SD), servings/d	2.51 (2.18)	2.49 (1.77)	2.79 (1.81)	3.13 (2.16)	2.88 (2.59)	2.51 (1.84)	2.74 (1.82)	2.98 (2.10)
Mean tea intake (SD), drinks/d	3.12 (3.88)	2.99 (3.77)	3.1 (3.52)	3.14 (3.59)	2.94 (3.90)	2.96 (3.82)	3.13 (3.55)	3.13 (3.61)
Variation in diet, n (%)	27,734 (58.88)	94,267 (65.63)	107,314 (66.79)	48,785 (65.33)	11,464 (56.85)	81,985 (65.61)	139,741 (65.99)	44,910 (64.87)

### Association of oily fish and nonoily fish intakes with all-cause mortality

In this study, 383,248 (88.9%) and 410,499 (95.2%) of the participants consumed oily fish and nonoily fish, respectively. Of the participants, 17.53% (n = 75,574) and 16.26% (n = 70,092) reported intakes of oily fish and nonoily fish, respectively, of more than 2 servings/week. During a median follow-up of 12.53 years, 25,899 deaths, including 12,870 cancer deaths and 5034 CVD deaths, were observed. The basic model was constructed with age and gender being adjusted, and we observed that the intakes of oily fish and nonoily fish were generally associated with lower risks of all-cause mortality, cancer mortality, and CVD mortality.

The results of the multivariable model demonstrated that, compared with the participants who never consumed oily fish, the participants reporting an oily fish consumption of < 1 or 1 serving/week had a lower risk of all-cause mortality. The adjusted HRs for the association of oily fish consumption (< 1 serving/week) and (1 serving/week) with all-cause mortality were 0.92 (0.86 to 0.98; *p <* 0.05) and 0.93 (0.87 to 0.98; *p <* 0.05), respectively. The intake of 1 serving/week of oily fish was associated with a lower risk of CVD mortality (HR = 0.85, 95% CI 0.74 to 0.98; *p <* 0.05) (Table 2).

**Table 2:** Associations of fish intake with all-cause and cause-specific mortality

Outcome	Never	Fish intake, hazard ratios (95% confidence interval)		
		**< **1 serving/week	1 serving/week	**≥ **2 serving/week
*Oily fish intake*				
All cause mortality				
Event, n (%)	3027 (6.33)	8086 (5.57)	9643 (5.93)	5143 (6.81)
Basic model^*^	1 (Reference)	0.82 (0.78–0.86)	0.79 (0.76–0.83)	0.85 (0.80–0.89)
Multivariable model^#^	1 (Reference)	0.92 (0.86–0.98)	0.93 (0.87–0.98)	0.98 (0.92–1.04)
Cancer mortality				
Event, n (%)	1383 (2.89)	4061 (2.80)	4986 (3.07)	2440 (3.23)
Basic model^*^	1 (Reference)	0.88 (0.82–0.94)	0.87 (0.81–0.93)	0.86 (0.80–0.93)
Multivariable model^#^	1 (Reference)	0.96 (0.89–1.05)	1.01 (0.92–1.10)	0.98 (0.89–1.08)
CVD mortality				
Event, n (%)	599 (1.25)	1588 (1.09)	1771 (1.09)	1076 (1.42)
Basic model^*^	1 (Reference)	0.81 (0.73–0.90)	0.74 (0.66–0.82)	0.90 (0.80–1.01)
Multivariable model^#^	1 (Reference)	0.93 (0.81–1.06)	0.85 (0.74–0.98)	1.02 (0.88–1.18)
*Nonoily fish intake*				
All cause mortality				
Event, n (%)	1279 (6.22)	7071 (5.59)	13,100 (6.12)	4449 (6.35)
Basic model^*^	1 (Reference)	0.91 (0.85–0.97)	0.93 (0.87–0.99)	0.98 (0.92–1.06)
Multivariable model^#^	1 (Reference)	0.93 (0.85–1.01)	0.96 (0.88–1.05)	1.02 (0.93–1.12)
Cancer mortality				
Event, n (%)	545 (2.65)	3576 (2.83)	6635 (3.10)	2114 (3.02)
Basic model^*^	1 (Reference)	1.05 (0.95–1.16)	1.07 (0.97–1.19)	1.07 (0.97–1.19)
Multivariable model^#^	1 (Reference)	0.99 (0.87–1.12)	1.01 (0.89–1.14)	1.03 (0.90–1.17)
CVD mortality				
Event, n (%)	242 (1.18)	1384 (1.09)	2551 (1.19)	857 (1.22)
Basic model^*^	1 (Reference)	0.95 (0.81–1.10)	0.97 (0.84–1.13)	1.00 (0.85–1.18)
Multivariable model^#^	1 (Reference)	0.95 (0.78–1.16)	1.02 (0.84–1.24)	1.05 (0.86–1.30)

Values were numbers (percentages) unless stated otherwise

CVD: cardiovascular disease

*Basic model: adjusted for baseline age and sex (male or female)

^#^Multivariable model: adjusted for age, sex (male or female), ethnic background (white or others), Townsend Deprivation Index, body mass index, physical activity (< 150 or ≥ 150 min/week), smoking status (current smoking or other), alcohol intake, processed meat intake, poultry intake, beef intake, lamb/mutton intake, pork intake, hypertension (yes or no), diabetes (yes or no), fish oil use (yes or no), fruit consumption, vegetables consumption, tea intake, variation in diet (yes or no). Oily fish and nonoily fish were mutually adjusted for each other

### Subgroup analysis

**Fig. 2:**
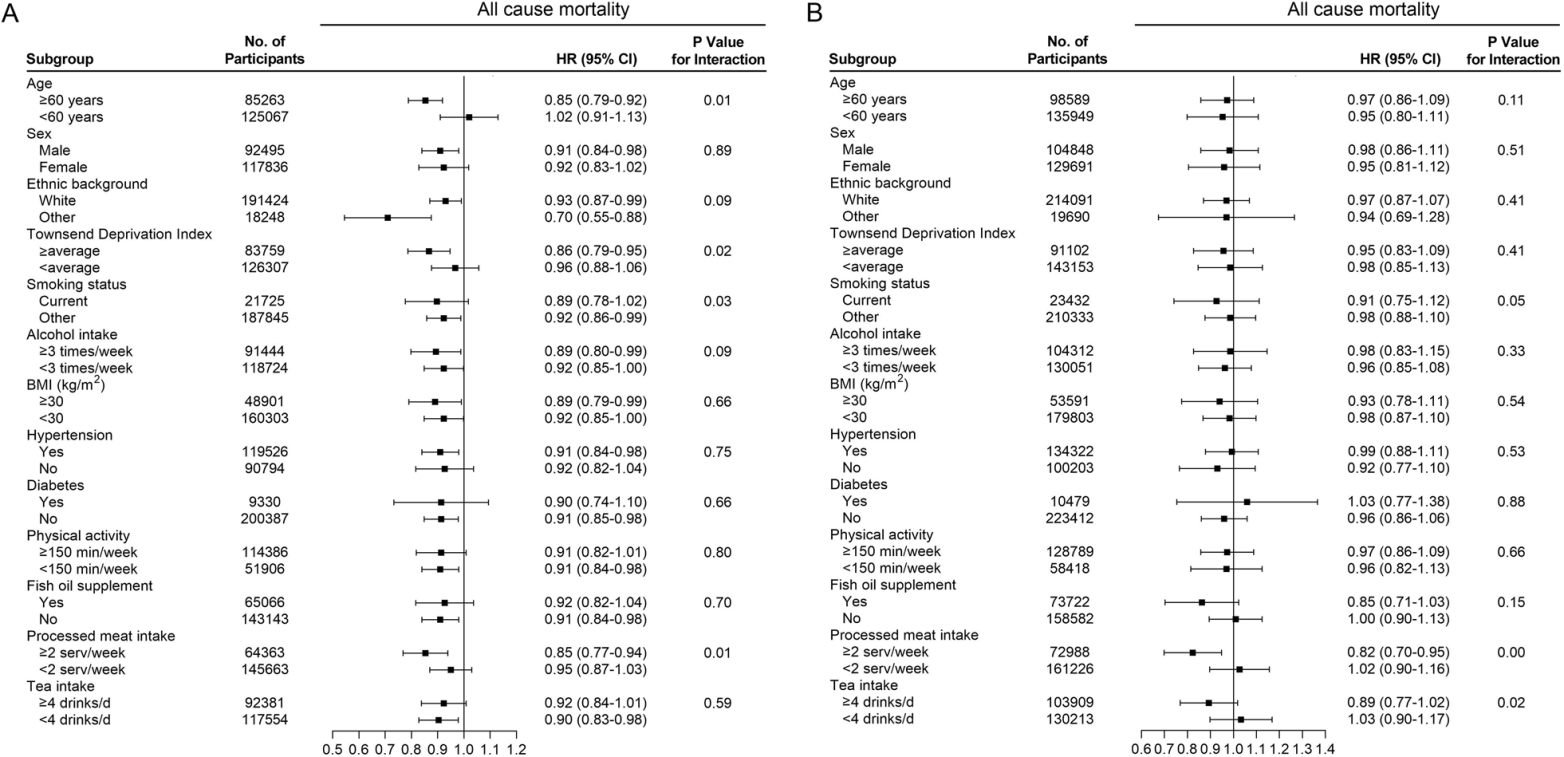
Association of **A** oily and **B** nonoily fish consumption and the risk of all cause mortality stratified by potential risk factors. The results were adjusted for age, sex (male or female), ethnic background (white or others), Townsend Deprivation Index, body mass index, physical activity (< 150 or ≥ 150 min/week), smoking status (current smoking or other), alcohol intake, processed meat intake, poultry intake, beef intake, lamb/mutton intake, pork intake, hypertension (yes or no), diabetes (yes or no), fish oil use (yes or no), fruit consumption, vegetables consumption, tea intake, variation in diet (yes or no). Oily fish and nonoily fish were mutually adjusted for each other

We performed several stratified analyses to evaluate the inverse associations of oily fish and nonoily fish intakes (1 serving/week) with all-cause mortality via a multivariable model. We observed that the consumption of oily fish was associated with all-cause mortality in the participants who were older than 60 years (HR = 0.85, 95% CI 0.79 to 0.92; *p <* 0.05), male (HR = 0.91, 95% CI 0.84 to 0.98; *p <* 0.05), those with a Townsend Deprivation Index score greater than or equal to the average score (HR = 0.86, 95% CI 0.79 to 0.95; *p <* 0.05), non-smoker (HR = 0.92, 95% CI 0.86 to 0.99; *p <* 0.05), those with higher alcohol intake frequency (HR = 0.89, 95% CI 0.80 to 0.99; *p <* 0.05), those with BMI ≥ 30 kg/m^2^ (HR = 0.89, 95% CI 0.79 to 0.99; *p <* 0.05), those with hypertension (HR = 0.91, 95% CI 0.84 to 0.98; *p <* 0.05), those without diabetes (HR = 0.91, 95% CI 0.85 to 0.98; *p <* 0.05), those with physical activity < 150 min/week (HR = 0.91, 95% CI 0.84 to 0.98; *p <* 0.05), non-user of fish oil (HR = 0.91, 95% CI 0.84 to 0.98; *p <* 0.05), those with an intake of processed meat of ≥ 2 servings/week (HR = 0.85, 95% CI 0.77 to 0.94; *p <* 0.05) and those with an intake of tea of < 4 drinks/day (HR = 0.90, 95% CI 0.83 to 0.98; *p <* 0.05) (Fig. 2A). Nonoily fish intake was associated with all-cause mortality in the those with an intake of processed meat of ≥ 2 servings/week (HR = 0.82, 95% CI 0.70 to 0.95; *p <* 0.05) (Fig. 2B).

**Fig. 3:**
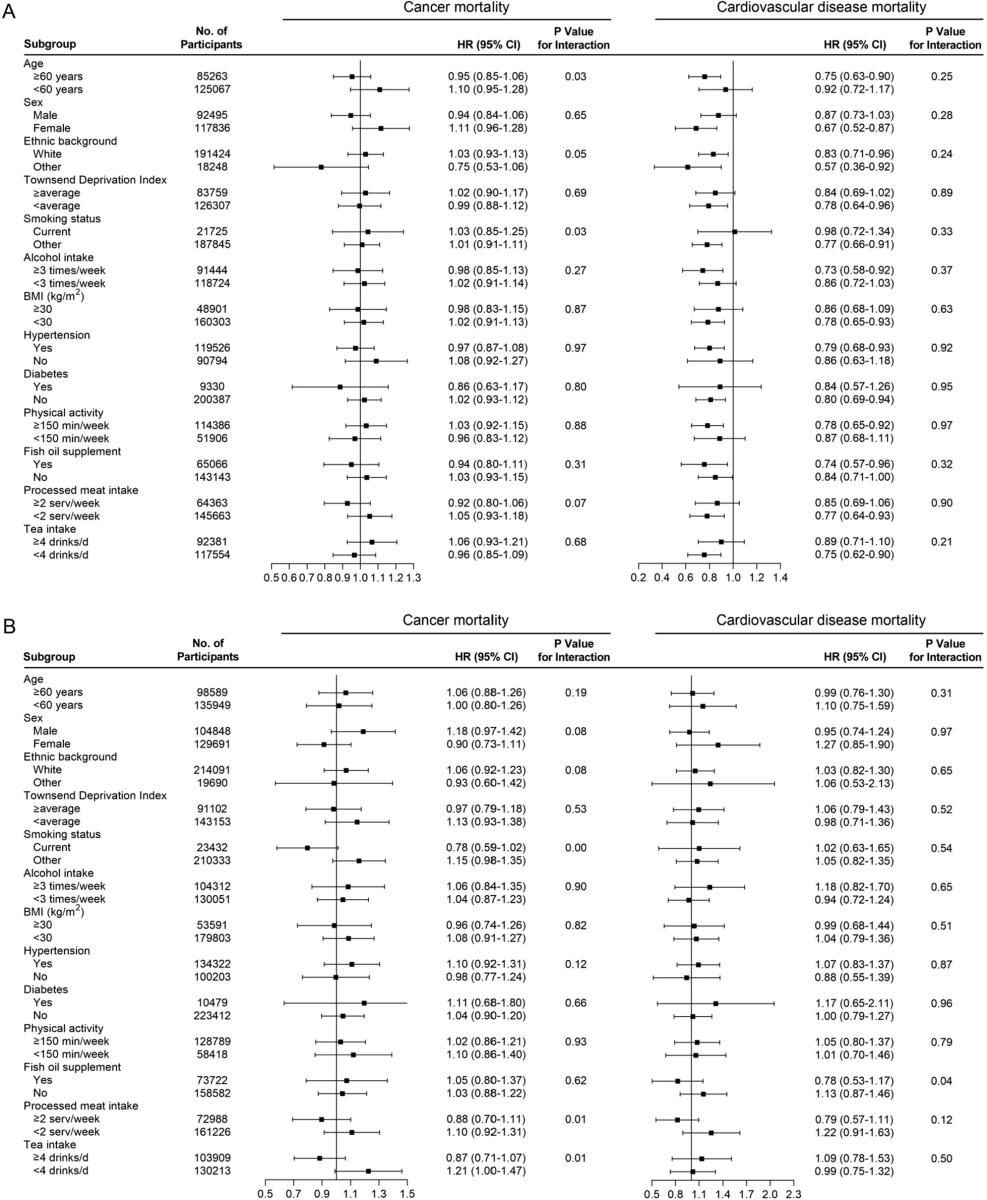
Associations of **A** oily and **B** nonoily fish consumption and the risk of cancer mortality and cardiovascular disease mortality stratified by potential risk factors. The results were adjusted for age, sex (male or female), ethnic background (white or others), Townsend Deprivation Index, body mass index, physical activity (< 150 or ≥ 150 min/week), smoking status (current smoking or other), alcohol intake, processed meat intake, poultry intake, beef intake, lamb/mutton intake, pork intake, hypertension (yes or no), diabetes (yes or no), fish oil use (yes or no), fruit consumption, vegetables consumption, tea intake, variation in diet (yes or no). Oily fish and nonoily fish were mutually adjusted for each other

For CVD mortality, oily fish intake was associated with a lower risk of CVD mortality in the participants who were older than 60 years, female, those with a Townsend Deprivation Index score less than the average score, non-smoker, those with higher alcohol intake frequency, those with BMI < 30 kg/m^2^, those with hypertension, those without diabetes, those with physical activity ≥ 150 min/week, fish oil user, those with an intake of processed meat of < 2 servings/week and those with an intake of tea of < 4 drinks/day (Fig. 3A). We observed stronger association between oily fish intake and all cause mortality and CVD mortality in non-white, where no significant interaction effect was found from the results of interaction analysis (Figs. 2A and 3A). No significant association was found in the subgroup analysis of nonoily fish (Fig. 3B).

Additionally, the inverse associations of oily fish intake with all-cause mortality were strongest in the participants who were older (*p* for interaction = 0.01) and non-smoker (*p* for interaction = 0.03). The association of nonoily fish intake with all-cause mortality was strongest in those with a processed meat intake of ≥ 2 servings/week *(p* for interaction = 0.00) (Fig. 2).

### Sensitivity analysis

 To determine the robustness of the results from the Cox proportional hazard models, we performed several sensitivity analyses. After excluding the participants who died during the first 2 years of follow-up from the models, the results did not change appreciably (Additional file 1: Table S2). Additionally, the results remained stable when we excluded participants who took vitamin or mineral supplements (Additional file 1: Table S3). Moreover, we observed that the results did not change significantly after excluding the participants with a diet that varied considerably from week to week (Additional file 1: Table S4).

## Discussion

UK Biobank is a large-scale biomedical database and research resource initiated by the UK government. The UK Biobank collected disease and lifestyle information and genotype data from approximately 500,000 participants aged 40 to 69 years from England, Scotland, or Wales between March 2006 and July 2010. The purpose of this project is to prevent, diagnose and treat many diseases, including cancer, heart disease, diabetes, and Alzheimer’s, so as to improve the health of the population. UK Biobank contained health information from half a million UK participants and conducted follow-up. Using the information collected by UK Bioabnk and the data from follow-up and physical examination, we were able to perform this study to explore the association of oily fish and nonoily fish intakes with all-cause mortality and cause-specific mortality.

In the present study of 431,062 participants without cancer or CVD at baseline, we observed that the intake of 1 serving/week of oily fish was associated with 7% and 15% lower risks of all-cause mortality and CVD mortality, respectively. The intakes of < 1 serving/week oily fish were associated with 8% lower risk of all-cause mortality. All of these relationships were independent of measures of potential confounders, including sociodemographic factors, socioeconomic status, lifestyle habits, health condition, and supplementation.

A previously published study demonstrated that the consumption of oily fish was associated with greater health benefits than the intake of other types of fish. Current dietary guidelines encourage the intake of various types of fish, preferably oily fish, including mackerel, tuna, sardines, and salmon [5, 25, 26]. The results of a large prospective cohort study suggested that regular fish oil supplementation was associated with lower risks of all-cause mortality and CVD outcomes. Additionally, the results of short-term clinical trials suggest that the intake of 2 servings/week of oily fish decreased triglyceride levels by 11.4% [27, 28]. Fish intake may lead to increased LDL-C levels, but this does not suggest that it leads to an increased risk of CVD, as this potential risk may be offset by the positive effect of oily fish intake on lipoprotein levels [29].

The inverse associations of oily fish intake with all-cause mortality and cause-specific mortality were strongest in the participants who were non-smokers. A previous study indicated that fish oil treatment could decrease the serum levels of the nitric oxide metabolites IL-1β, IL-6, and TNF-α [30]. An oily fish diet may improve subclinical inflammation [31]. The potential reason for observing a stronger protective effect in non-smokers is that these smokers were in a state of lower inflammatory stress at baseline. The association between oily fish intake and all-cause mortality was strongest in the participants with a BMI of ≥ 30 kg/m^2^. An explanation for this may be that the consumption of oily fish helps reduce lipid levels in people with obesity, so this consumption may provide more benefit to lowering the risk of all-cause mortality in these individuals.

Additionally, our study demonstrates that oily fish intake is associated with lower risks of all-cause mortality and CVD mortality with a moderate intake of oily fish (1 serving/week). The risk of all-cause mortality and CVD mortality did not further decrease with a higher consumption of oily fish, which is a result that is similar to the results of a previous study [32].

## Strengths and limitations

There are some strengths of this study, such as the minimal loss to follow-up, large-scale design, and prospectively population-based cohort design. At the same time, our study has several limitations. First, although potential confounders were adjusted for in this study, we cannot completely ruled out residual confounding. Second, this study was an observational study. Thus, causality could not be determined. Third, although we evaluated oily fish and nonoily fish separately, but did not obtain information on more-specific types of fish and thus did not analyze their associations with mortality. Fourth, most consumers of oily fish and consumers of nonoily fish in this study had low to moderate levels of fish intake, which limits the generalizability of the findings of our study to populations with high oily fish and nonoily fish intakes. Fifth, the participants in our study were mainly from the UK, which may limit the generalizability of our conclusions to populations outside of the UK.

## Conclusions

A total of 431,062 participants without cancer or CVD at baseline were included in the present study. Of these participants, 383,248 (88.9%) and 410,499 (95.2%) were consumers of oily fish and nonoily fish, respectively. Compared with those who did not consume fish, those with an oily fish intake of 1 serving/week exhibited lower risks of all-cause mortality and CVD mortality. Collectively, our study suggests that the consumption of 1 serving/week of oily fish is recommended for improving clinical outcomes.

## Supplementary Information

**Additional file 1: Table S1.** ACE touchscreen question for fish consumption in the UK Biobank. **Table S2.** Associations of fish intake with risk of all-cause and cause-specific mortality after excluding participants who experienced death during the first two years of follow-up.** Table S3.** Associations of fish intake with risk of all-cause and cause-specific mortality after excluding participants who took vitamin and mineral supplements. **Table S4.** Associations of fish intake with risk of all-cause and cause-specific mortality after excluding participants with diet varying much from week to week.

## Data Availability

The datasets used and/or analyzed during the current study are available from the corresponding author upon reasonable request.
