# Supplementary material for: Association of oily fish and nonoily fish intakes with all-cause mortality and cause-specific mortality: a large population-based prospective study
Source: J Transl Med. 2023 Apr 26;21:280. doi: 10.1186/s12967-023-04097-4 (PMC10131441; doi:10.1186/s12967-023-04097-4)
Supplement: Supplementary file 1 — Additional file 1: Table S1. ACE touchscreen question for fish consumption in the UK Biobank. Table S2. Associations of fish intake with risk of all-cause and cause-specific mortality after excluding participants who experienced death during the first two years of follow-up. Table S3. Associations of fish intake with risk of all-cause and cause-specific mortality after excluding participants who took vitamin and mineral supplements. Table S4. Associations of fish intake with risk of all-cause and cause-specific mortality after excluding participants with diet varying much from week to week. [file 12967_2023_4097_MOESM1_ESM.docx]

| **Fish intake** | **ACE touchscreen question** | **Coding and meaning** | **Help button** |
| --- | --- | --- | --- |
| Oily fish | How often do you eat oily fish? (e.g. sardines, salmon, mackerel, herring) | 0: Never  1: Less than once a week  2: Once a week  3: 2-4 times a week  4: 5-6 times a week  5: Once or more daily  -1: Do not know  -3: Prefer not to answer | **Please provide an average considering your intake over the last year.** If you are unsure, please provide an estimate or select Do not know. **Oily fish include:**   1. Salmon Anchovies 2. Trout Swordfish 3. Mackerel Bloater 4. Herring Cacha 5. Sardines Carp 6. Pilchards Hilsa 7. Kipper Jack fish 8. Eel Katla 9. Whitebait Orange roughy 10. Tuna (fresh only) Pangas 11. Sprats |
| Nonoily fish | How often do you eat other types of fish? (e.g. cod, tinned tuna, haddock) | 0: Never  1: Less than once a week  2: Once a week  3: 2-4 times a week  4: 5-6 times a week  5: Once or more daily  -1: Do not know  -3: Prefer not to answer | **Please provide an average considering your intake over the last year.** If you are unsure, please provide an estimate or select Do not know. |

Table S1. ACE touchscreen question for fish consumption in the UK Biobank.

Table S2. Associations of fish intake with risk of all-cause and cause-specific mortality after excluding participants who experienced death during the first two years of follow-up.

| **Outcome** | **Never** | **Fish intake, hazard ratios (95% confidence interval)^#^** | | |
| --- | --- | --- | --- | --- |
|  |  | <1 serving/week | 1 serving/week | ≥2 serving/week |
| **Oily fish intake** |  |  |  |  |
| All cause mortality | 1 (Reference) | 0.91 (0.85-0.97) | 0.92 (0.86-0.98) | 0.96 (0.90-1.03) |
| Cancer mortality | 1 (Reference) | 0.96 (0.88-1.05) | 1.00 (0.92-1.09) | 0.97 (0.88-1.07) |
| CVD mortality | 1 (Reference) | 0.93 (0.81-1.07) | 0.86 (0.75-0.99) | 1.02 (0.87-1.18) |
| **Nonoily fish intake** |  |  |  |  |
| All cause mortality | 1 (Reference) | 0.93 (0.85-1.02) | 0.96 (0.88-1.05) | 1.03 (0.94-1.13) |
| Cancer mortality | 1 (Reference) | 0.99 (0.87-1.13) | 1.01 (0.89-1.14) | 1.02 (0.89-1.17) |
| CVD mortality | 1 (Reference) | 0.96 (0.78-1.18) | 1.02 (0.83-1.26) | 1.06 (0.86-1.32) |

CVD=cardiovascular disease.

^#^ multivariable model: adjusted for age, sex (male or female), ethnic background (white or others), Townsend Deprivation Index, body mass index, physical activity (<150 or ≥150 min/week), smoking status (current smoking or other), alcohol intake, processed meat intake, poultry intake, beef intake, lamb/mutton intake, pork intake, hypertension (yes or no), diabetes (yes or no), fish oil use (yes or no), fruit consumption, vegetables consumption, tea intake, variation in diet (yes or no). Oily fish and nonoily fish were mutually adjusted for each other.

Table S3. Associations of fish intake with risk of all-cause and cause-specific mortality after excluding participants who took vitamin and mineral supplements.

| **Outcome** | **Never** | **Fish intake, hazard ratios (95% confidence interval)^#^** | | |
| --- | --- | --- | --- | --- |
|  |  | <1 serving/week | 1 serving/week | ≥2 serving/week |
| **Oily fish intake** |  |  |  |  |
| All cause mortality | 1 (Reference) | 0.90 (0.84-0.97) | 0.90 (0.84-0.97) | 0.93 (0.86-1.00) |
| Cancer mortality | 1 (Reference) | 1.00 (0.90-1.11) | 1.04 (0.94-1.16) | 0.97 (0.87-1.09) |
| CVD mortality | 1 (Reference) | 0.88 (0.75-1.02) | 0.76 (0.64-0.88) | 0.91 (0.76-1.08) |
| **Nonoily fish intake** |  |  |  |  |
| All cause mortality | 1 (Reference) | 0.93 (0.83-1.03) | 0.99 (0.89-1.10) | 1.05 (0.94-1.18) |
| Cancer mortality | 1 (Reference) | 0.96 (0.82-1.12) | 0.98 (0.84-1.14) | 1.00 (0.85-1.17) |
| CVD mortality | 1 (Reference) | 1.11 (0.86-1.43) | 1.30 (1.01-1.67) | 1.30 (1.00-1.69) |

CVD=cardiovascular disease.

^#^ multivariable model: adjusted for age, sex (male or female), ethnic background (white or others), Townsend Deprivation Index, body mass index, physical activity (<150 or ≥150 min/week), smoking status (current smoking or other), alcohol intake, processed meat intake, poultry intake, beef intake, lamb/mutton intake, pork intake, hypertension (yes or no), diabetes (yes or no), fish oil use (yes or no), fruit consumption, vegetables consumption, tea intake, variation in diet (yes or no). Oily fish and nonoily fish were mutually adjusted for each other.

Table S4. Associations of fish intake with risk of all-cause and cause-specific mortality after excluding participants with diet varying much from week to week.

| **Outcome** | **Never** | **Fish intake, hazard ratios (95% confidence interval)^#^** | | |
| --- | --- | --- | --- | --- |
|  |  | <1 serving/week | 1 serving/week | ≥2 serving/week |
| **Oily fish intake** |  |  |  |  |
| All cause mortality | 1 (Reference) | 0.86 (0.79-0.95) | 0.91 (0.83-1.00) | 0.95 (0.86-1.06) |
| Cancer mortality | 1 (Reference) | 0.88 (0.77-1.01) | 0.97 (0.85-1.11) | 0.91 (0.79-1.05) |
| CVD mortality | 1 (Reference) | 0.97 (0.79-1.20) | 0.87 (0.69-0.99) | 1.00 (0.79-1.26) |
| **Nonoily fish intake** |  |  |  |  |
| All cause mortality | 1 (Reference) | 0.93 (0.82-1.06) | 0.98 (0.86-1.11) | 1.00 (0.87-1.15) |
| Cancer mortality | 1 (Reference) | 1.06 (0.87-1.28) | 1.08 (0.9-1.31) | 1.06 (0.87-1.3) |
| CVD mortality | 1 (Reference) | 0.97 (0.71-1.31) | 1.02 (0.76-1.38) | 1.02 (0.74-1.41) |

CVD=cardiovascular disease.

^#^ multivariable model: adjusted for age, sex (male or female), ethnic background (white or others), Townsend Deprivation Index, body mass index, physical activity (<150 or ≥150 min/week), smoking status (current smoking or other), alcohol intake, processed meat intake, poultry intake, beef intake, lamb/mutton intake, pork intake, hypertension (yes or no), diabetes (yes or no), fish oil use (yes or no), fruit consumption, vegetables consumption, tea intake, variation in diet (yes or no). Oily fish and nonoily fish were mutually adjusted for each other.
